# Resistance Status of the Malaria Vector Mosquitoes, *Anopheles stephensi* and *Anopheles subpictus* Towards Adulticides and Larvicides in Arid and Semi-Arid Areas of India

**DOI:** 10.1673/031.011.8501

**Published:** 2011-07-07

**Authors:** S. N. Tikar, M.J. Mendki, A. K. Sharma, D. Sukumaran, Vijay Veer, Shri Prakash, B. D. Parashar

**Affiliations:** Division of Entomology, Defence R&D Establishment, Jhansi Road, Gwalior, MP- 474002, India

**Keywords:** insecticide, toxicity, Rajasthan, Gujarat, Punjab, *Anopheles*

## Abstract

Susceptibility studies of malaria vectors *Anopheles stephensi* Liston (Diptera: Culicidae) and *An. subpictus* Grassi collected during 2004–2007 from various locations of Arid and Semi-Arid Zone of India were conducted by adulticide bioassay of DDT, malathion, deltamethrin and larvicide bioassay of fenthion, temephos, chlorpyriphos and malathion using diagnostic doses. Both species from all locations exhibited variable resistance to DDT and malathion from majority of location. Adults of both the species were susceptible to Deltamethrin. Larvae of both the Anopheline species showed some evidence of resistance to chlorpyriphos followed by fenthion whereas susceptible to temephos and malathion.

## Introduction

Malaria is a major global health problem. The estimated 247 million malaria cases with almost half of the global population at risk and nearly a million deaths each year (WHO 2009). Among the 109 malaria endemic countries, India had 1.5 million confirmed malaria cases in 2009 with over 1,000 deaths (WHO 2010). Several Anopheles species are responsible for transmission of malaria. *Anopheles stephensi* Liston (Diptera: Culicidae) and *An. subpictus* Grassi are commonly found during our survey work in Arid and Semi arid zone of Rajasthan and Gujarat. *An. stephensi* is a sub-tropical species distributed throughout the Middle East and South Asia and is a major vector of malaria in urban areas in India accounting for about 12% of malaria cases annually and also is an important malaria vector in Pakistan and Iran (Dash et al. 2007). This species perennially transmits malaria, is an important vector in arid zones of Rajasthan where it has a unique characteristic of breeding proficiently in underground water tanks prevalent in villages and urban areas. (Dash et al. 2006).

*An. subpictus* is another species that is widely distributed in oriental regions and is a prolific breeder in most parts of India during the rainy season. Sibling species A of *An. subpictus* (fresh water form) has been incriminated and established as a primary vector of malaria in Tarakeswar, West Bengal (Chatterjee and Chandra 2000). In Orissa, this species was incriminated as a vector of malaria in 2009 (Kumari et al. 2009). *An. subpictus*, is the major malaria vector in the Jaffna area and is a well-established secondary vector of malaria in other part of Srilanka (Kannathasan et al. 2008). Japanese encephalitis virus in India has been isolated from 16 mosquito species including *An. subpictus* (Samule et al. 2000). This species has been reported to be resistant to DDT and dieldrin/HCH in Gujarat (NMEP 1991).

Transmission of malaria can be reduced by adopting vector control measures such as indoor residual spraying with insecticides, larval control measures and personal protection measures. The combination of tools and methods used to combat malaria now includes insect nets treated with long lasting insecticides and artemisinin-based combination therapy, supported by indoor residual spraying of insecticide and intermittent preventive treatment during pregnancy. Among these, indoor residual spraying has been the main method of mosquito control in India covering about 80 million households and protecting 40% of the population at risk (WHO 2008). Currently 12 insecticides are recommended by WHO for indoor spraying. In India, the main insecticides used for indoor residual spraying include DDT, malathion and synthetic pyrethroids in rural areas and source reduction and anti-larval measure in urban areas. However, continuous use of targeted insecticides has led to the development of resistance in many malaria vectors around the world. In India several anopheline species have become resistant to insecticides. *An. culicifacies*, which is the main malaria vector in India, responsible for 60–70% of malaria cases, has been shown to be resistant to DDT and malathion in India (Dash et al. 2006). This rural vector was not encountered during mosquito collection in our study.

In the present study locations, spraying for mosquito control was done regularly, however, resistance levels in malaria vectors has not been monitored for any insecticides. Therefore, the present study was done to determine the susceptibility status of *Anopheles* adults and larval stages to the recommended insecticides. The information generated will ensure the pattern of insecticide use that is necessary in these areas avoids increased insecticide use that could threaten the sustainability of the vector control strategy by causing increased resistance. Thus, monitoring of insecticide susceptibility/resistance status against malaria vectors *An. stephensi* and *An. subpictus* in arid and semi-arid areas will generate data that will be helpful in future insecticide resistance management strategies targeted against malaria vectors in these regions.

**Figure 1.**
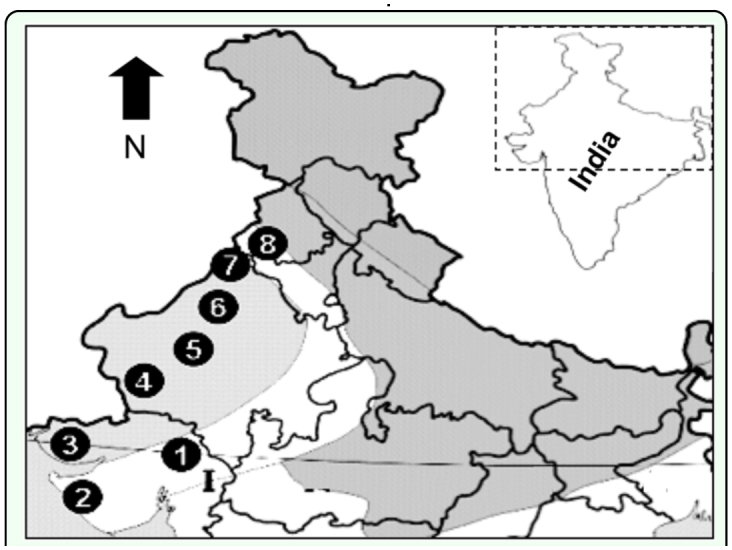
Mosquito collection sites. 1. Gandhinagar 2. Jamnagar 3. Bhuj 4. Barmer 5. Jodhpur 6. Bikaner 7. Sriganganagar 8. Bathinda. High quality figures are available online.

## Materials and Methods

### Test Insects

Mosquitoes were collected from different cantonment areas belonging to arid and semiarid regions (Table 1, Figure 1) *An. stephensi* larvae were collected from breeding sites such as water storage tanks, fountains, pipe leakages, whereas *An. subpictus* larvae were collected mainly from muddy water, from ponds, stagnant water channel and rainwater collections.

**Table 1.**
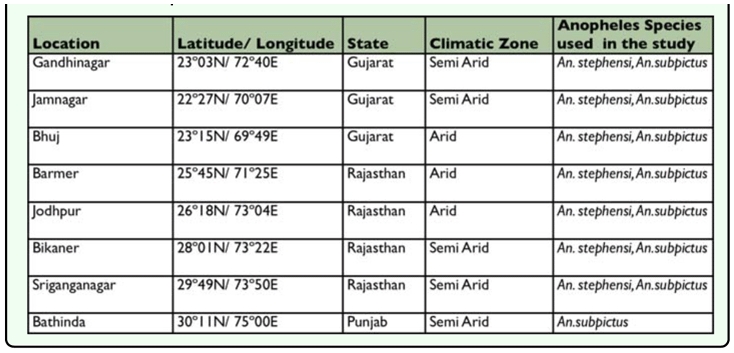
Mosquito collection sites.

### Insecticides

Technical grade insecticides used in the present study were deltamehtrin 98.42% and temephos 90.63% provided by Heranba Chemicals (www.heranba.co.in), fenthion 99.9% was purchased from Riedel-de-Haen, (www.riedeldehaen.com), malathion 96% and DDT p,p isomer 77% were gifts of the Hindustan Insecticide Ltd., (www.hindustaninsecticides.com) whereas chlorpyrifos 99% was from Bharat Rasayan, (www.bharatgroup.co.in).

### Adult bioassay

Anopheline adult mosquitoes were collected from the study locations between 1800–2000 hrs using glass mouth aspirators and held in cages and fed with 10% sugar solution *ad libitum* dispensed through a cotton wick. In certain cases, when insufficient field collected adults were obtained, females (3–5 days old) emerged from field collected larvae were also used for adult bioassay. The standard test for determining insecticide resistance in adult mosquitoes was conducted on field caught mosquitoes using diagnostic doses (WHO 1981). Rectangular pieces of Whatman paper measuring 12 cm×15 cm were impregnated with 2 ml mixture of acetone and a non volatile carrier, olive oil for OP insecticides and DDT, and silicon oil for pyrethroids. The final concentration of the oil applied was 3.6mg/cm^2^ paper. The impregnation was done by pipetting solution evenly onto the filter paper. The papers were then air dried and stored until use. During bioassay 20 female mosquitoes were exposed to the diagnostic doses of DDT (4.0%), malathion (5.0%), and deltamethrin (0.05%) for 1 hour and transferred in a separate cage provided with 10% sugar solution and mortality observations were made after 24 hours. The WHO criterion was followed for considering the vector species susceptible (mortality>98 %), resistant (mortality <80 %) and tolerant/intermediate resistant (mortality 80 – 98 %). The number of females exposed varied from 20–40 in different study locations. Knockdown time for deltamethrin was monitored every 4 min. interval during the 1 hr. exposure and the time required for 50% knockdown of mosquitoes (KD50) was determined using probit analysis (Finney 1971) using statistical software. The same bioassays were carried out on the laboratory-reared susceptible *An. stephensi* strain to compare the susceptibility levels of the field populations.

**Table 2.**
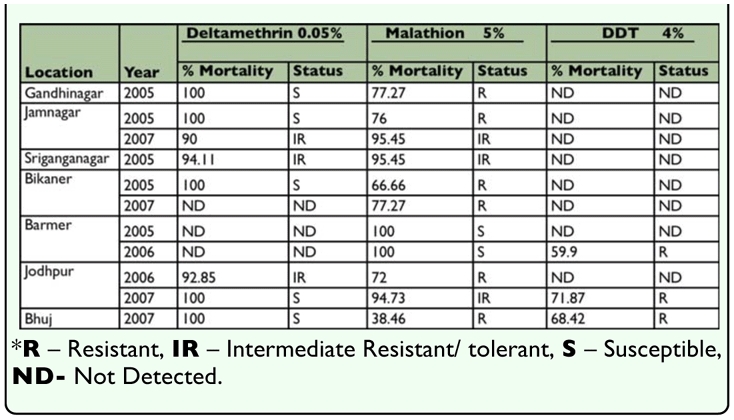
Adult bioassay of Deltamethrin, Malathion and DDT to *Anopheles stephensi* (2005–2007).

**Table 3.**
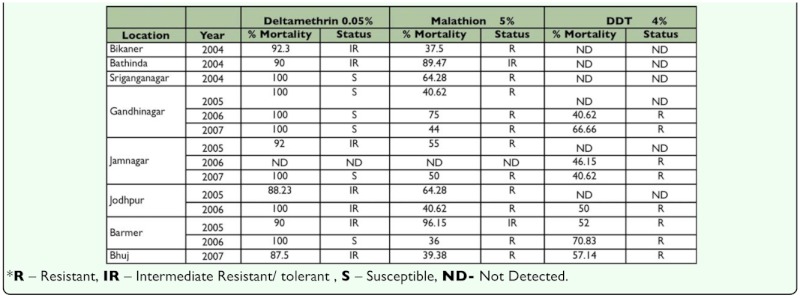
Adult bioassay of Deltamethrin, Malathion and DDT to *Anopheles subpictus* (2004–2007).

### Larval Bioassay

The larval susceptibility to insecticides assay was carried out according to method of WHO (1981). Field collected late third to early fourth instar larvae were sorted out and 25 larvae were transferred into disposable 200 ml plastic cups containing 99 ml of dechlorinated tap water. One milliliter of insecticide solution of diagnostic dose was dispensed with a micropipette in the water cup. Dried brewers yeast powder was given as larval diet. Larval mortality was recorded after 24 hr. Moribund larvae (presenting tremors, rigidity or inability to reach water surface on touch) were considered as dead. The experiment was replicated twice. Test was not rejected for control mortality <20 % or when pupation was 10%.

## Results and Discussion

Adult bioassay of deltamethrin, malathion and DDT to *An. stephensi* shows that adults collected from all the locations during 2005–07 were susceptible to or tolerant to deltamethrin 0.05% with a mortality range of 92.85–100% (Table 2). *An. stephensi* adults collected from Gandhinagar, Jamnagar and, Bikaner during 2005, Jodhpur during 2006, Bhuj and Bikaner during 2007 exhibited varied levels of resistance to 5% malathion, (adult mortality 38.46–77.27%), whereas they were susceptible from Banner during both years. DDT resistance was found in adults collected from all the three locations tested; Barmer, Jodhpur and Bhuj.

**Table 4.**
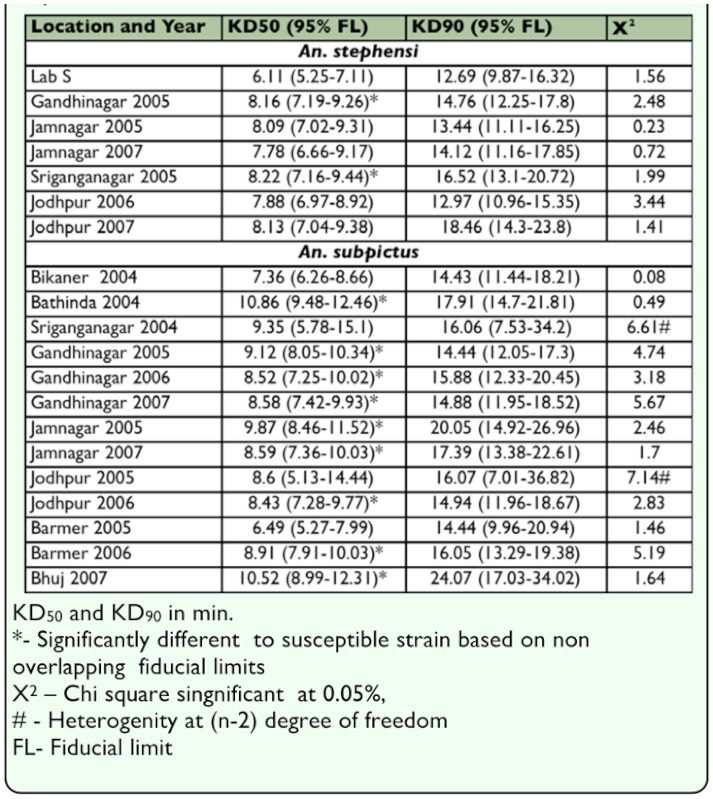
Knockdown Bioassay of *Anopheles stephensi* and *An*. *subpktus* to deltamethrin 0.05 %.

Adult bioassay of deltamethrin, malathion and DDT to *An. subpictus* collected from all the locations during 2004–07 (Table 3) were susceptible or tolerant to 0.05% deltamethrin (Table 3) with a mortality range of 88.23–100%. Malathion resistance was reported for *An. subpictus* from the majority of the locations studied with adult mortality of 36–75% except Bathinda and Barmer where they were tolerant to 5% malathion. *An. subpictus* was found resistance to DDT from all the eight locations tested (adult mortality 40.62–70.83%).

The knockdown bioassay of 0.05 % deltamethrin to *An. stephensi* (Table 4), KD_50_ of 6.11 min was determined for laboratory susceptible strain of *An. stephensi* that was compared to that of the field population. Significantly higher values KD_50_ of 8.16 and 8.22 min from Gandhinagar and Sriganganagar was observed whereas for rest of the locations KD_50_ ranged from 7.78–8.13 min. Overall from all locations calculated KD_50_ was below 1.5 times the KD_50_ of the susceptible *An. stephensi* laboratory strain. Knockdown bioassay of 0.05 % deltamethrin to *An. subpictus* adults (Table 4) collected from various field locations was in the range of 6.49–10.52 min.

**Table 5.**
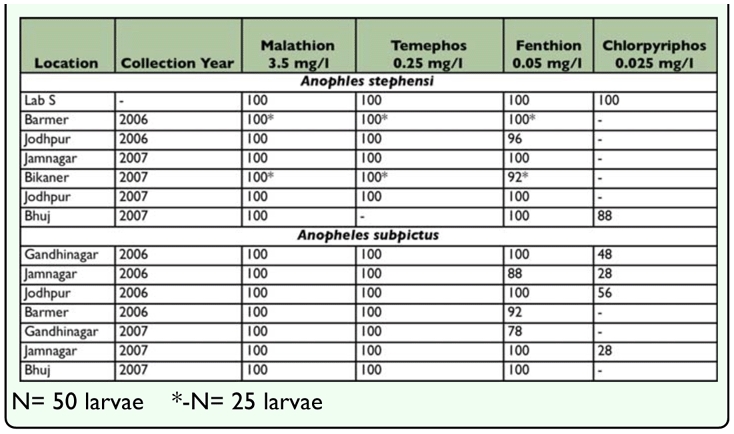
Larval Bioassay of Insecticides (% Larval Mortality).

Larval bioassay of insecticides to larvae of *An. stephensi* (Table 5) revealed that field collected larvae were susceptible to larvicides at diagnostic doses of malathion (3.5 mg/l), temephos (0.25 mg/l) and fenthion (0.05 mg/l). *An. subpictus* larvae were also susceptible to malathion, temephos and fenthion at diagnostic doses from all locations except from Gandhinagar where 78% larval mortality to fenthion 0.05 mg/l was reported. Larvae of *An. stephensi* were tolerant to chlorpyriphos 0.025 mg/l (mortality of 88 %) from Bhuj whereas *An. subpictus* exhibited chlorpyriphos resistant from Gandhinagar Jamnagar, Jodhpur and Bhuj with larval mortality of 28–56%.

In India, widespread insecticide resistance has been reported in the major malaria vectors *An. stephensi* and *An. culicifacies*. In spite of several reports on insect vector resistance to DDT, it is inexpensive and has a very good repellency activity and the longest residual efficacy. DDT is used for 60–65% internal residual spraying in India followed by synthetic pyrethroid and malathion.

In the present study, higher levels of DDT resistance was observed in *An. stephensi* collected from all the locations studied ie. Barmer, Jodhpur and Bhuj, similar type of studies on DDT resistance in *An. stephensi* was also reported from Jodhpur, Barmer, Jaisalmer and the Bikaner area of the Thar desert (Batra et al. 1999; Singh and Bansal 2006 2007; Bansal and Singh 1996, Singh and Bansal 1996) from Eastern portion of India i.e. Culcutta (Mukhopadhyay et al. 1996) from Western India i.e. Goa (Thavaselvam et al. 1993) and from Pakistan (Rathor et al. 1980). On the contrary, *An. stephensi* from Mangalore were recently found to be susceptible to DDT (Tiwari et al. 2010). Even though DDT and HCH are not directly used against this vector in urban areas, their use in periurban and rural areas has induced resistance in this species, however in rural areas, *An. stephensi* is not a serious vector and therefore its resistance to residual insecticides is not a problem for malaria control. In urban areas, control of *An. stephensi*—induced malaria is primarily dependent on antilarval methods and indoor spraying of insecticides (Mittal et al. 2004). One possibility for the reduced susceptibility of *An. quadriannulatus* to DDT could be selection in larval stages (Mzilahowa et al. 2008). In the present study, variable DDT resistance in *An. subpictus* was observed in Gandhinagar, Jamnagar, Jodhpur and Barmer. A similar report of resistance from Bikaner (Bansal and Singh 1996) has been reported. Recently very high levels of DDT resistance (adult mortality 14–47%) and malathion resistance (adult mortality 23–49%) in *An. subpictus* was reported from five districts of Sri Lanka (Parera et al. 2008).

The widespread phenomenon of resistance in vectors was one of the three main factors that contributed to ineffectiveness of DDT in India (Sharma 2003). The Stockholm convention on the persistent use of organic pollutants has an exemption for the production and public health use of DDT for indoor application to control vector-borne diseases, mainly because of the absence of equally effective and efficient alternatives (WHO 2007). WHO suggested no change to its current recommendations on the safety of DDT for disease vector control, with the continuous monitoring of the status of insecticide resistance in order to select insecticides to which vectors are susceptible and also for implementation of resistance management tactics (WHO 2007).

We observed malathion resistance in *An. stephensi* and *An. subpictus* from the majority of the locations studied. The high level of malathion resistance in several locations is probably a direct result of malathion used for mosquito control. Malathion resistance in *An. culicifacies* was first reported from Gujarat in 1973, and later became widespread throughout the country. Malathion resistance in *An. stephensi* was reported from Surat and Gujarat (MRC/STP 1999) Jodhpur, Barmer, and Jaisalmer (Singh and Bansal 2006 2007) and recently from Mangalore (Tiwari et al. 2010). Partial resistance was reported from Barmer, Jodhpur and Pali (Singh and Bansal 1996), from Culcutta (Mukhopadhyay et al. 1996) and from Goa (Thavaselvam et al. 1993). Distribution of sibling species, agricultural pesticides and/or other environmental factors are possibly responsible for widespread malathion resistance in *An. subpictus* from some part of Srilanka (Kelly-Hope et al. 2005). Higher levels of malathion resistance could be due to agricultural pesticides used for paddy pest control where *An. subpictus* breeds as well as the use of pesticides for malarial control (Herath and Joshi 1989).

In this study, both Anopheline species were found susceptible to deltamethrin and the knockdown time was not much different from that of the susceptible strain. NVBDCP advocates use of synthetic pyrethroids where Anophelines are resistant to DDT as well as to malathion. In India synthetic pyrethroids were introduced in public health programs in the 1990s to combat a malaria epidemic and to control triple-resistant mosquitoes in certain localities (Singh et al. 2002). Synthetic pyrethroids are being used in public health programs to control multiple-resistant vectors and tackle epidemic outbreaks. Also, this is the only group of insecticides currently used for bed-net impregnation for malaria control. In addition, commonly synthetic pyrethroids are used for vector control via mosquito coils, mats and liquid vaporizers. A significant decline in positivity rate and reduction in the incidence of malaria was observed due to deltamethrin—impregnated mosquito nets (Joshi et al. 2003). In India, synthetic pyrethroids and organophosphate pesticides are currently being used in not only for vector control, but also in the agricultural sector, mainly for control of lepidopteron pests.

In the present study, both Anopheline species were effectively killed at diagnostic doses of fenthion and temephos. However, a larval population of *An. subpictus* from Andhra Pradesh showed a high degree of resistance to fenthion and temephos (Sharma et al. 2003).

In general, chlorpyriphos is not used commonly for *An. subpictus* larval control. One of the reasons why chlorpyriphos resistance in *An. subpictus* is observed might be due to its use in agricultural pest control. Chlorpyriphos is used in rice paddies during the transplanting stage for pest control. Kant et al. (1992) found that *An. culicifacies* and *An. subpictus* were dominant in newly transplanted fields during early months of rice cultivation.

Variation in insecticide resistance mainly depends upon the type of insecticide and frequency of use. Excessive and unwanted usage of insecticides not only increases vector resistance, but also results in cross resistance to other insecticides. Although various mechanisms of insecticide resistance in insects such as metabolic resistance (i.e. esterases, monooxigenase or glutathione-s-transferase), resistance due to reduced penetration or behavioural resistance are reported in several vectors, generally it is governed by either involvement of metabolic mechanisms or alterations at target sites. Revealing the mechanism of resistance is equally important to that of monitoring resistance in mosquito vectors. Overall, in the present study, it was found that both the Anopheline species are highly resistant to DDT as well as moderately resistant to malathion but are susceptible to deltamethrin from majority of the locations studied. Insecticide resistance is a serious emerging problem in India. Currently, the national program has no alternative insecticide for effective vector control or for insecticide resistance management (Dash et al. 2006). Since there are limited numbers of insecticides available for vector control, an approach focused on the rotational use of insecticides or a mosaic strategy can be adopted to delay development of resistance in malaria vectors as studied in a field trial in Mexico to manage multi-insecticide resistant *An. albimanus* (Rodriguez et al. 2006). Also, emphasis needs to be given to other ecofriendly methods of vector control, such as biocontrol with larvivorous fish and biolarvicides especially *Bacillus thuringiensis* var. *israelensis* included in the integrated vector management program (Ghosh and Dash 2007; Tiwari et al. 2011). Insecticides are currently most practical in controlling mosquito vector, and therefore cannot be overlooked. Effective resistance management mainly depends upon early detection of the status of resistance, therefore monitoring of insecticide resistance at regular intervals is necessary so that an effective management strategy can be designed.

## References

[bibr01] Bansal SK, Singh KV (1996). Insecticide susceptibility status of some anophelines in district Bikaner, Rajasthan.. *Indian Journal of Malariology*.

[bibr02] Batra CP, Mittal PK, Adak T, Sharma VP (1999). Malaria investigations in District Jodhpur Rajasthan, during the summer season.. *Indian Journal of Malariology*.

[bibr03] Chatterjee SN, Chandra G (2000). Role of *Anopheles subpictus* as aprimary vector of malaria in an area in India.. *Japanese Journal of Tropical Medicine and Hygiene*.

[bibr04] Dash AP, Adak T, Raghavendra K, Singh OP (2007). The biology and control of malaria vectors in India.. *Current Science*.

[bibr05] Dash AP, Raghavendra K, Pillai MKK (2006). Combating Resistance to Insecticides in Malaria Control- Gains Made in India.. *Bayer Environmental Science Journal*.

[bibr06] Finney J.D. (1971). *Probit analysis*.

[bibr07] Ghosh SK, Dash AP (2007). Larvivorous fish against malaria vectors: a new outlook.. *Transactions of the Royal Society of Tropical Medicine and Hygiene*.

[bibr08] Herath PR, Joshi GP (1989). Pesticide selection pressure on *Anopheles subpictus* in Sri Lanka: comparison with two other Sri Lankan anophelines.. *Transactions of Royal Society of Tropical Medicine and Hygiene*.

[bibr09] Joshi RM, Ghose G, Som TK, Bala S (2003). Study of the impact of deltamethrin impregnated mosquito nets on malaria incidence at a military station.. *Medical Journal Armed Forces India*.

[bibr10] Kannathasan A, Antonyrajan KA, Srikrishnaraj A, Karunaratne SHPP, Karunaweera ND, Surendran SN (2008). Studies on prevalence of anopheline species and community perception of malaria in Jaffna district, Sri Lanka.. *Journal of Vector Borne Diseases*.

[bibr11] Kant R, Pandey SD, Sharma RC (1992). Seasonal prevalence and succession of rice field breeding mosquitoes of central Gujarat.. *Journal of Communicable Diseases*.

[bibr12] Kelly-Hope LA, Yapabandara AM, Wickramasinghe MB, Perera MD, Karunaratne SH, Fernando WP, Abeyasinghe RR, Siyambalagoda RR, Herath PR, Galappaththy GN, Hemingway J (2005). Spatiotemporal distribution of insecticide resistance in *Anopheles culicifacies* and *Anopheles subpictus* in Sri Lanka.. *Transaction of Royal Society of Tropical Medicine and Hygiene*.

[bibr13] Kumari S, Parida SK, Marai N, Tripathy A, Hazra RK, Kar SK, Mahapatra N (2009). Vectorial role of *Anopheles subpictus* grassi and *Anopheles culicifacies* giles in Angul district, Orissa, India..

[bibr14] Mittal PK, Wijeyaratne P, Sabeena P (2004). *Status of insecticide resistance of malaria, Kala - azar and Japanese encephalitis vector in Bangladesh, Bhutan, India and Nepal.* Prepared under EPH Project 26568/E.X.ANE.MRCCOORE..

[bibr15] MRC/STP (1999). *Annual report of science & technology projection integrated vector control of Malaria Research Centre.*.

[bibr16] Mukhopadhyay AK, Chakraborty S, Karmakar PK, Banerjee P (1996). Insecticidal susceptibility status of *Anopheles stephensi* (Liston) in selected areas of Calcutta (West Bengal).. *Indian Journal of Public Health*.

[bibr17] Mzilahowa T, Ball AJ, Bass C, Morgan JC, Nyoni B, Steen K, Donnelly MJ, Wilding CS (2008). Reduced susceptibility to DDT in field populations of *Anopheles quadriannulatus* and *Anopheles arabiensis* in Malawi: evidence for larval selection.. *Medical Veterinary Entomology*.

[bibr18] NMEP (1991). *Annual Report of the National Malaria Eradication Programme*.

[bibr19] Perera MD, Hemingway J, Karunaratne SP (2008). Multiple insecticide resistance mechanisms involving metabolic changes and insensitive target sites selected in anopheline vectors of malaria in Sri Lanka.. *Malaria Journal*.

[bibr20] Rathor HR, Toqir G, Reisen WK (1980). Status of insecticide resistance in anopheline mosquitoes of Punjab Province, Pakistan.. *Southeast Asian Journal of Tropical Medicine and Public Health*.

[bibr21] Rodriguez AD, Penilla RP, Rodriguez H, Hemingway J (2006). Insecticide resistance management in a multi-resistant malaria vector scenario: A Mexican trial shows sustainability.. *Bayer Environmental Science Journal*.

[bibr22] Samuel PP, Hiriyan J, Gajanana A (2000). Japanese encephalitis virus infection in mosquitoes and its epidemiological implications.. *ICMR Bulletin*.

[bibr23] Sharma RS, Sharma SN, Kumar A (2003). Susceptibility status of Japanese encephalitis vectors in Kurnool and Mehboobnagar districts of Andhra Pradesh, India.. *Journal of Communicable Diseases*.

[bibr24] Sharma VP (2003). DDT: The fallen angel.. *Current Science*.

[bibr25] Singh KV, Bansal SK (2007). Mapping of insecticide resistance in vectors of malaria in Rajasthan.. http://dmrcjodhpur.org/AR06-07/p1-7.pdf.

[bibr26] Singh KV, Bansal SK (2006). Mapping of insecticide resistance in vectors of malaria in Rajasthan..

[bibr27] Singh KV, Bansal SK (1996). Current status of *Anopheles stephensi* response to various insecticides in some areas of the Thar Desert.. *Indian Journal of Medical Research*.

[bibr28] Singh OP, Raghavendra K, Nanda N, Mittal PK, Subbarao SK (2002). Pyrethroid rsistance In *Anopheles culicifacies* In Surat District, Gujarat, West India.. *Current Science*.

[bibr29] Thavaselvam D, Kumar A, Sumodan PK (1993). Insecticide susceptibility status of *Anopheles stephensi*, *Culex quinquefasciatus* and *Aedes aegypti* in Panaji. Goa.. *Indian Journal of Malariology*.

[bibr30] Tiwari SN, Ghosh SK, Ojha VP, Dash AP, Raghavendra K. (2010). Reduced susceptibility to selected synthetic pyrethroids in urban malaria vector *Anopheles stephensi*: a case study in Mangalore city, South India.. *Malaria Journal.*.

[bibr31] Tiwari SN, Ghosh SK, Mittal PK, Dash AP (2011). Effectiveness of a granular formulation of *Bacillus thuringiensis* var. *israelensis* against larvae of malaria vectors in India.. *Vector-Borne Zoonotic Diseases*.

[bibr32] WHO (2010). *World malaria report.*.

[bibr33] WHO (2009). *10 facts on malaria*..

[bibr34] WHO 2008 World malaria report.

[bibr35] WHO (2007). *The Use of DDT In Malaria Vector Control WHO position statement.*.

[bibr36] WHO (1981). *Criteria and meaning of tests for determining the susceptibility or resistance of insects to insecticides*.

